# Preferential Nitrogen and Phosphorus Reallocation to Apical Needles Drives Basal Needle Chlorosis in *Pinus sylvestris* L. Plantations in the Otindag Sandy Land

**DOI:** 10.3390/biology15070518

**Published:** 2026-03-24

**Authors:** Xu Zhang, Chengzhen Jia, Bailing Miao, Yongli Wang, Cunzhu Liang

**Affiliations:** 1 Key Laboratory of Ecology and Resource Use of the Mongolian Plateau, Ministry of Education of China, School of Ecology and Environment, Inner Mongolia University, Hohhot 010021, China; bilcz@imu.edu.cn; 2 Inner Mongolia Meteorological Institute, Hohhot 010051, China; jcz6011@outlook.com (C.J.); miaobailing@126.com (B.M.)

**Keywords:** nutrient reallocation, ecological stoichiometric, needle chlorosis, sandy-land plantation

## Abstract

Leaf yellowing seriously affects the sustainability of artificial forest ecosystems. However, it remains unclear whether such chlorosis is driven primarily by soil nutrient deficiency or by internal nutrient reallocation. In particular, the physiological processes underlying the green apices and yellow bases pattern within branches remain poorly understood. This study compared needle carbon (C), nitrogen (N), and phosphorus (P) stoichiometry between apical and basal positions in asymptomatic and symptomatic *Pinus sylvestris* L. trees within the Otindag Sandy Land, China. Our findings revealed that except for the 80–100 cm layer, soil element concentrations did not differ significantly between healthy and chlorotic trees. In the trees, apical needles maintained stable stoichiometry across all trees, whereas basal needles of symptomatic individuals exhibited significantly higher C:N and C:P ratios, indicating severe localized nutrient stress. Notably, symptomatic trees exhibited exceptionally high N and P resorption efficiencies (79.68% and 71.05%, respectively), which were significantly higher than those of healthy trees (41.73% and 48.09%). The high Stoichiometric Deviation Index (SDI) and weak needle–soil correlations further confirm that needle chlorosis is decoupled from direct soil supply limitations. Instead, this pattern is primarily governed by prioritized internal nutrient reallocation to safeguard apical growth dominance. These findings highlight branch-level nutrient redistribution as a useful adaptive strategy to consider when interpreting early decline symptoms and nutrient stress in sandy-land *P. sylvestris* plantations.

## Simple Summary

Pine forests planted in sandy regions are crucial for preventing desertification, but many are experiencing severe decline, often visible as the yellowing of older pine needles. Our study aimed to understand the exact cause of this yellowing in Scots pine trees. We discovered that this color change is not simply a sign of the tree passively starving due to poor soil. Instead, it is a highly active survival strategy. That tree deliberately extracts these valuable elements from its older, lower needles and moves them to support the fresh, growing needles at the top. In short, the tree sacrifices its older leaves to ensure its most vital parts survive under severe stress. This discovery helps society better monitor the health of degrading plantations and develop more effective conservation and management strategies to protect these plantations from climate change and continued desertification.

## 1. Introduction

Afforestation is crucial for restoring degraded sandy ecosystems, providing key services such as windbreak, sand fixation, and soil improvement [1,2]. Despite these benefits, sandy ecosystems are typically characterized by chronic nutrient poverty and strong resource limitation, which can undermine plantation sustainability and lead to tree decline and dieback [3,4]. Notably, even within the same plantation, leaf condition can diverge sharply despite shared soil and climate conditions. Some individuals maintain uniformly green leaves across the entire crown. In contrast, others display a distinctive branch-level pattern where basal leaves become chlorotic while apical leaves remain green. This suggests that leaf chlorosis may not be a simple soil nutrient deficiency but rather a physiological adjustment within the plant [5]. However, it remains unclear whether leaf chlorosis in sandy ecosystems is caused by soil nutrient deficiency or by internal nutrient regulation within the trees.

Conventionally, leaf chlorosis is attributed to soil nutrient deficiencies, as the disruption of chlorophyll synthesis and chloroplast function is a direct physiological consequence of an inadequate supply of essential mineral elements, particularly nitrogen (N) and phosphorus (P) [6,7,8]. Firstly, N is a fundamental structural component of chlorophyll molecules and proteins involved in photosynthesis. Under N limitation, chlorophyll production declines, leading to a reduction in the green pigmentation of leaves. The plant may also remobilize N from older leaves to support new growth, exacerbating chlorosis in mature needles [9,10]. Secondly, while not a direct component of chlorophyll, P is essential for energy transfer and membrane integrity. Phosphorus deficiency can impair energy metabolism and the synthesis of key organic compounds, indirectly stifling chlorophyll production and leading to a dull, dark-green, or purplish hue that can progress to chlorosis, particularly in older leaves [11,12]. Leaf chlorosis appears to be driven not only by external soil nutrient availability but also by complex internal nutrient reallocation dynamics. For example, weak or negative correlations between leaf and soil N and P contents have been widely observed in *Pinus sylvestris* L. and *Populus alba* L. plantations in the Horqin Sandy ecosystem [13,14], as well as in the Hengduan Mountains [15]. However, such common relationships do not imply a complete absence of external soil influence. A more nuanced interpretation suggests a partial physiological decoupling: under chronic soil nutrient limitation, trees increasingly rely on internal nutrient cycling to sustain essential metabolic functions. Therefore, the specific basal leaves chlorotic pattern observed within a single branch is likely not a simple, passive symptom of soil poverty. Instead, it results from an active internal reallocation of nutrients—particularly N and P—triggered by severe environmental stress. Such remobilization from senescing to active tissues is a fundamental plant strategy to cope with stress or imbalanced supply [16,17]. When demand exceeds uptake, plants may reallocate nutrients from older, senescing tissues to support younger, high-value sinks such as apical meristems—a strategy aligned with Optimal Partitioning Theory [18,19]. Nevertheless, within the chronically nutrient-depauperate context of sandy ecosystems, it remains unresolved whether branch basal chlorosis is primarily a passive consequence of soil nutrient deficiency or a manifestation of a proactive internal reallocation strategy triggered by environmental stress.

The Otindag Sandy Land, a key ecological barrier in northern China, has undergone extensive afforestation through initiatives like the Three-North Shelterbelt Forest Program [20]. Despite these long-term restoration efforts, branch basal needle chlorosis has become exceptionally widespread in *P. sylvestris* plantations across this chronically nutrient deficiency region. This widespread decline highlights the challenge of sustainable plantation management and provides an ideal context to investigate the specific physiological drivers of needle chlorosis.

This study investigates healthy and chlorotic *P. sylvestris* plantations within the Otindag sandy ecosystem. The specific objectives of this research were to: (1) compare the C, N, and P concentrations, alongside their stoichiometric ratios, between apical and basal needles across both tree conditions; (2) evaluate the intensity and specific priority of internal nutrient reallocation; and (3) determine the extent to which needle stoichiometry is coupled with, or decoupled from, soil nutrient availability. We hypothesize that branch basal needle chlorosis is primarily driven by a nutrient reallocation strategy triggered to safeguard apical growth dominance, rather than by direct soil nutrient deficiency. By uncovering the internal nutrient allocation patterns behind needle chlorosis, this study offers a novel perspective for understanding plantation forest decline and mortality in sandy land ecosystems.

## 2. Materials and Methods

### 2.1. Study Area

**Figure 1:**
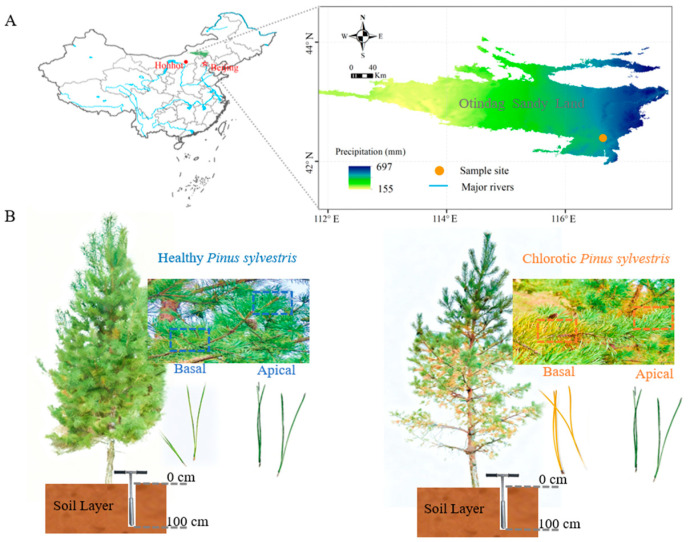
Sampling sites and tree conditions (healthy vs. chlorotic) in *P. sylvestris* plantations on the Otindag Sandy Land. (**A**) The location and range of the Otindag Sandy Land; (**B**) Healthy and chlorotic *P. sylvestris* trees.

Located at 115.87° E–117.07° E and 41.75° N–42.65° N, the Otindag Sandy Land covers an area of 53,000 km^2^ (Figure 1A). It is the sandy land closest to Beijing, with a straight-line distance of merely 180 km. The terrain of the Otindag Sandy Land is higher in the southwest and lower in the northeast, with an average elevation of 1300 m. The mean annual temperature ranges from 1.8 °C to 3.2 °C, and the annual precipitation ranges from 250 to 400 mm, decreasing from the southeast to the northwest. The region is dominated by aeolian sandy soils, which are intrinsically characterized by severe nutrient deficiency and poor structural stability. Previous broad-scale surveys in the Otindag Sandy Land have demonstrated that the soil organic carbon (SOC), total N, and total P are exceptionally low, averaging approximately 2.30, 0.23, and 0.13 mg/g, respectively [21]. The region experiences a temperate continental climate. From east to west, the vegetation types transition sequentially through savanna, shrubland, and desert steppe. The native vegetation primarily includes the tree species *Ulmus pumila* L., as well as shrubs such as *Caragana microphylla* L. and herbaceous plants including *Eulaliopsis binata* R., *Stipa baicalensis* R., and *Artemisia frigida* W. The *P. sylvestris* is the main plantation tree species in this area.

### 2.2. Sample Collection

The study was conducted in a *P. sylvestris* plantation established in 2011. Trees were selected based on visual symptom assessment: (1) chlorotic trees exhibiting chlorosis needles at branch bases but green needles at apices; (2) healthy trees with no visible discoloration. Although the initial tree selection relied on visual assessment, the chlorotic phenotype in this specific plantation was highly severe and unambiguous. There was a stark, binary visual contrast between the completely yellow basal needles and the dark green apical needles on the same symptomatic branches, minimizing the risk of subjective misclassification. All trees were of similar age (15 years) and DBH (15–20 cm). Needle samples were collected in September 2025 from the sunny aspects of the crowns. Fine-scale intra-branch sampling and deep soil profile coring (down to 100 cm) are highly intensive and destructive. Therefore, to minimize stand impact, three healthy trees and three chlorotic trees were selected as biological replicates. For each chlorotic tree, needles were separately collected from the basal chlorosis section and the apical green section. To strictly avoid pseudoreplication, needles from various branches of a single tree were homogenized. Consequently, only one composite sample was analyzed per position per tree. Correspondingly, needles from healthy branches of the healthy trees were sampled from comparable positions. Samples were immediately frozen in liquid nitrogen and later oven-dried for nutrient analysis (Figure 1B). For each tree (healthy and chlorotic), soil was sampled within a 50 cm distance from the trunk. At each sampling point, a soil core was obtained down to 100 cm depth with a soil auger and subsequently partitioned into five layers (0–20, 20–40, 40–60, 60–80, and 80–100 cm) [5,13]. All soil material from the same depth layer across sampling points was homogenized to create a composite sample per depth per tree. Samples were sieved and stored at −20 °C.

In the laboratory, needle samples were dried at 65 °C until constant weight, then ground using a ball mill and passed through a 2 mm sieve for subsequent analysis. Soil samples were air-dried, after which gravel and roots were removed. They were then ground and sieved through a 2 mm mesh for chemical analysis. Total carbon (C), N, and P concentrations in needles and soil were determined following standardized chemical protocols [22]. Total C was measured via the potassium dichromate (K_2_Cr_2_O_7_) oxidation method with external heating and titration with ferrous sulfate. Total N was determined using the semi-micro Kjeldahl method, involving digestion with concentrated H_2_SO_4_ and subsequent distillation using an automated unit (Kjeltec™ 8400, Foss, Hillerød, Denmark). Total P was analyzed colorimetrically using the ammonium molybdate blue method; plant samples were digested with H_2_SO_4_−H_2_O_2_, while soil samples underwent NaOH fusion. The absorbance of the phosphomolybdenum blue complex was measured at 700 nm using a UV−2700 spectrophotometer (Shimadzu, Kyoto, Japan).

### 2.3. Statistical Analyses

The ratios of carbon to nitrogen (C:N), carbon to phosphorus (C:P), and nitrogen to phosphorus (N:P) were calculated for both needle and soil samples. The degree of stoichiometric homeostasis for a given ratio in different branch positions was assessed using the Homeostatic Index (*HI*), calculated as the inverse of the coefficient of variation (*CV*) following the method of Su and Shangguan [23]:

$$HI\;=\;1/CV\;=\;\bar{X}/SD$$

where $\bar{X}$ is the mean and *SD* is the standard deviation of the stoichiometric ratio across samples within a defined group. A higher *HI* value indicates stronger homeostatic regulation (less variability), while a lower *HI* value suggests a breakdown in homeostasis.

A Nutrient Stress Index (*NSI*) was developed to integrate the information from both N and P status [13]:

*NSI* = Z_(*C:N*)_ + Z_(*C:P*)_

Here, *Z_(C:N)_* and *Z_(C:P)_* are the Z−scores of the standardized values of the needle C:N and C:P ratios, respectively, calculated across all needle samples. This index quantifies the overall degree of nutrient limitation, with more positive values indicating greater combined N and P stress.

Nutrient resorption efficiency (*NRE*, %) prior to needle senescence was estimated for nitrogen and phosphorus using the concentration difference between mature and senescing needles on the same symptomatic branch, according to the formula [18]:

*NRE_Nutrient_* = (*C_Apical_* − *C_Basal_*)/*C_Apical_* × 100%

where *C_Apical_* and *C_Basal_* represent the nutrient concentration (N or P) in the apical and basal needles of a branch, respectively. A higher *NRE* value indicates a greater proportion of the nutrient was withdrawn from the basal senescing tissue.

To determine the preferential resorption between N and P, a Resorption Priority Index (*RPI*) was calculated [24]:

*RPI* = *NRE_N_*/*NRE_P_*

An *RPI* > 1 indicates preferential resorption of N over P, *RPI* ≈ 1 indicates coupled resorption, and *RPI* < 1 indicates preferential resorption of P.

To evaluate the decoupling between needle stoichiometry and soil nutrient balance, a Stoichiometric Deviation Index (*SDI*) was calculated [13]:

*SDI* = |(*Needle_Ratio_*) − (*Soil_Ratio_*)_corresponding_|/(*Soil_Ratio_*)_corresponding_

where (*Soil_Ratio_*)_corresponding_ is the corresponding stoichiometric ratio measured in the soil. The larger the *SDI* value indicates the smaller impact of soil nutrient balance on needle stoichiometry. Pearson correlation analysis was also used to examine the relationships between needle and soil stoichiometry.

Moreover, Principal Component Analysis (PCA) is used to examine the differences in the ratio of needle stoichiometry. Differences in elemental stoichiometry between groups were examined using analysis of variance (ANOVA). Prior to ANOVA, the homogeneity of variances was tested using Levene’s test. For data with homogeneous variances, one-way ANOVA followed by Tukey’s HSD post hoc test was performed. For data with heterogeneous variances, Welch’s ANOVA was applied. Differences in the RPI between healthy and chlorotic trees were evaluated using a *t*-test. All statistical analyses and visualizations were performed using Origin 2025b software. The significance level for all tests was set at *p* = 0.05.

## 3. Results

### 3.1. Needle and Soil Stoichiometry Characteristic

**Figure 2:**
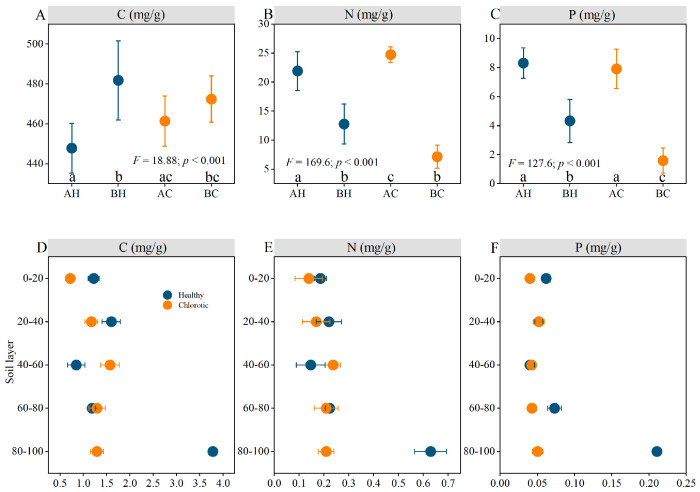
Element concentrations of needles (**A**–**C**) and soil (**D**–**F**) in *P. sylvestris* plantations on the Otindag Sandy Land. AH = the apical needle of healthy tree; BH = the basal needle of healthy tree; AC = the apical needle of chlorotic tree; BC = the basal needle of chlorotic tree. Data are presented as mean ± standard deviation (*n* = 3). Different lowercase letters denote significant differences at *p* < 0.05. The *F* and *p* values represent the statistical parameters from the one-way ANOVA.

Stoichiometric characteristics differed obviously between apical and basal needles of the branch (Figure 2A–C). In healthy trees, the mean concentrations of N and P in apical needles were 21.9 and 8.3 mg/g, respectively, which were significantly higher than those in basal needles (12.8 and 4.3 mg/g). Similarly, in chlorotic trees, the N and P concentrations in apical needles (24.7 and 7.9 mg/g) were significantly higher than those in basal needles (7.2 and 1.6 mg/g). In contrast to N and P, the C concentrations in apical needles of both healthy and chlorotic trees (447.8 and 461.4 mg/g, respectively) were lower than those in their corresponding basal needles (481.8 and 472.4 mg/g). Notably, although apical needle C and P concentrations were similar between healthy and chlorotic trees, the N concentration was slightly higher in chlorotic trees. Significant differences in the soil stoichiometry characteristics were only observed in the 80–100 cm soil layer between healthy and chlorotic trees. No significant differences were found in the other soil layers (Figure 2D–F).

**Figure 3:**
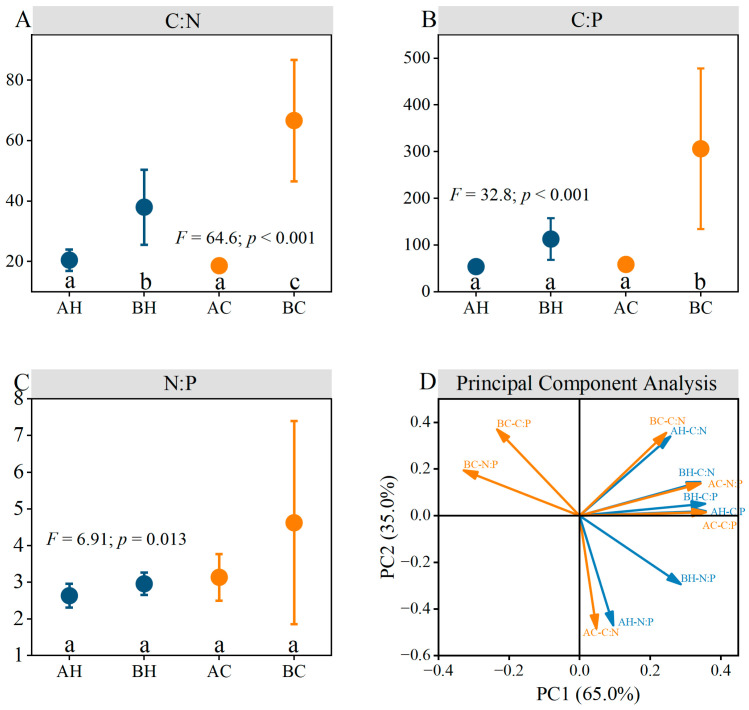
Stoichiometric characteristic ratio (**A**–**C**) and Principal Component Analysis (**D**) of needles in *P. sylvestris* plantations on the Otindag Sandy Land. AH = the apical needle of healthy tree; BH = the basal needle of healthy tree; AC = the apical needle of chlorotic tree; BC = the basal needle of chlorotic tree. C:N = the ratio of total carbon to total nitrogen; C:P = the ratio of total carbon to total phosphorus; N:P = the ratio of total nitrogen to total phosphorus. Data are presented as mean ± standard deviation (*n* = 3). Different lowercase letters denote significant differences at *p* < 0.05. The *F* and *p* values represent the statistical parameters from the one-way ANOVA.

A key finding was that the C:N and C:P in the branch basal needles of chlorotic trees were significantly higher than in apical needles and in the healthy trees (Figure 3A,B). This result suggests that the basal needle chlorosis in chlorotic trees is consistent with severe shortages of both N and P. Furthermore, although not statistically significant, the higher N:P ratio observed in the basal needle of chlorotic trees provides corroborative evidence for this result (Figure 3C). Similarly, the C:P and N:P at the branch bases of chlorotic trees primarily account for the variance in the second principal component (Figure 3D). This pattern further suggests that needle chlorosis is associated with nitrogen and phosphorus limitations.

### 3.2. Internal Nutrient Reallocation and Homeostasis of Needles

**Figure 4:**
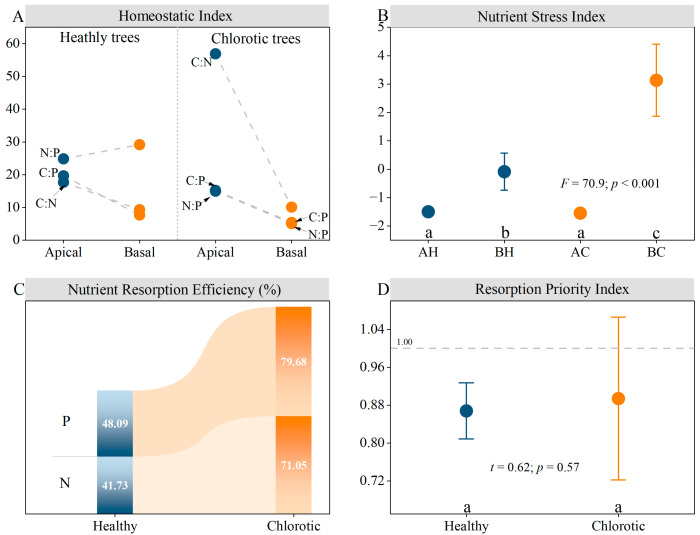
Key nutrient indices of needles in *P. sylvestris* plantations on the Otindag Sandy Land. (**A**) the Homeostatic Index; (**B**) the Nutrient Stress Index; (**C**) the Nutrient resorption efficiency; (**D**) the Resorption Priority Index. N = total nitrogen; P = total phosphorus; C:N = the ratio of total carbon to total nitrogen; C:P = the ratio of total carbon to total phosphorus; N:P = the ratio of total nitrogen to total phosphorus. Different lowercase letters denote significant differences at *p* < 0.05. The *F* and *p* values represent the statistical parameters from the one-way ANOVA. The *t* values represent the statistical parameters from the *t*-test.

Significant differences in *HI* were observed between branch apical and basal needles for all stoichiometric ratios except for the N:P ratio in healthy trees (Figure 4A). Notably, the *HI* for the C:N was obviously higher in apical needles than in basal, particularly in chlorotic trees. This suggests a highly stable nutrient status in branch apical needles. Furthermore, the *NSI* was consistently higher in branch basal needles compared to apical in both healthy and chlorotic trees, suggesting that basal needles experience stronger nutrient limitation, especially in chlorotic trees (Figure 4B). This pattern was further substantiated by the significantly higher *NRE* of N and P in chlorotic trees compared to healthy ones (Figure 4C). The higher *NRE* suggests a substantial remobilization of N and P from branch basal needles to support apical growth. Moreover, the *RPI* was approximately one for both healthy and chlorotic trees, suggesting that N and P were translocated concurrently to the branch apical needles (Figure 4D).

### 3.3. The Relationship Between Needle and Soil Element Stoichiometry Characteristics

**Figure 5:**
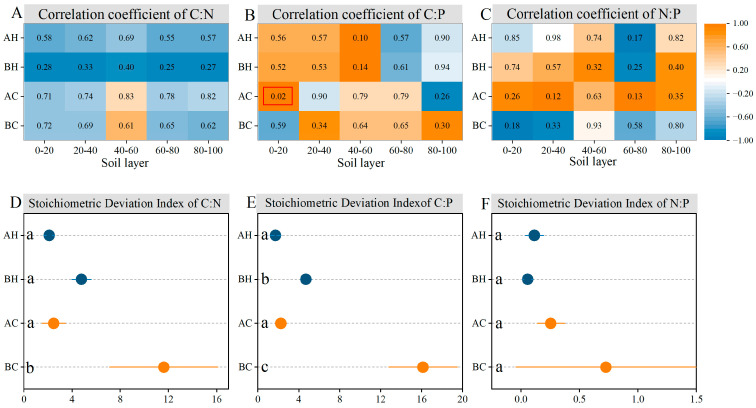
Correlation coefficient (**A**–**C**) and Stoichiometric Deviation Index (**D**–**F**) between needle and soil elemental stoichiometry characteristics in *P. sylvestris* plantations on the Otindag Sandy Land. Numbers in the correlation plot represent *p–*values, and red boxes indicate statistical significance at the *p* < 0.05 level. AH = the apical needle of healthy tree; BH = the basal needle of healthy tree; AC = the apical needle of chlorotic tree; BC = the basal needle of chlorotic tree. Different lowercase letters denote significant differences at *p* < 0.05.

Correlation analysis revealed that only the C:P ratio in the 0–20 cm soil layer showed a significant positive correlation with branch apical needles of chlorotic trees, while no significant correlations were observed in other soil layers (Figure 5A–C). This suggests that the needle nutrient status was largely decoupled from the soil nutrient availability. Concurrently, the higher *SDI* values observed for the basal needles of chlorotic trees further demonstrate that the nutrient status of the needle chlorosis was largely decoupled from the soil nutrient conditions (Figure 5D–F).

## 4. Discussion

### 4.1. The Reallocation of N and P to Branch Apical Needles Resulted in Basal Needle Chlorosis

**Figure 6:**
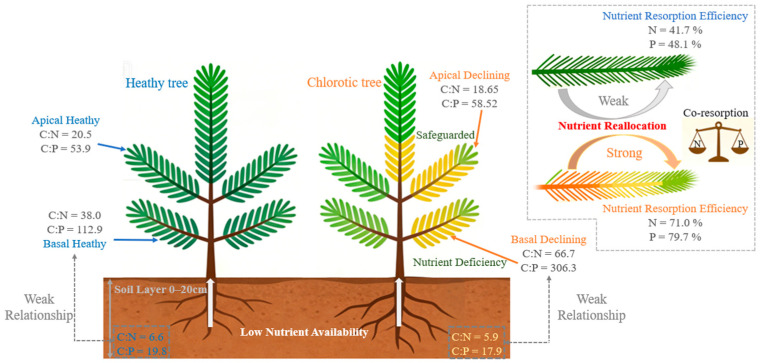
Schematic diagram of needle chlorosis in *P. sylvestris* plantations on the Otindag Sandy Land. N = total nitrogen; P = total phosphorus; C:N = the ratio of total carbon to total nitrogen; C:P = the ratio of total carbon to total phosphorus; N:P = the ratio of total nitrogen to total phosphorus.

Our study supports the hypothesis that branch basal needle chlorosis results from the reallocation of N and P to apical needles. The basal needle chlorosis of chlorotic trees is in a state of extreme co–limitation by both N and P. In the Otindag Sandy Land of our study area, soil C:N and C:P were below 7 and 20, respectively—significantly lower than the global averages for forest and wildland soils (14.3 and 186) [25] and also below the mean values for surface soils in China (11.9 and 61) [26]. This suggests a pronounced nutrient deficiency in the study region. However, combining analysis of healthy trees suggests that the phenomenon is not attributable to insufficient soil supply but to a reallocation of nutrients within the tree (Figure 6). When perceiving certain stresses (e.g., drought, low temperature, or competitive stress), the plant adopts a survival strategy that sacrifices older needles to prioritize apical growth [27,28]. This strategy manifests as exceptionally high N and P resorption efficiencies, leading to abnormal stoichiometric signatures—specifically higher C:N and C:P—in the basal needles.

This finding can be interpreted through the Stoichiometric Homeostasis Theory and the Relative Resorption Hypothesis. The former posits that plants maintain a relatively stable internal nutrient composition despite environmental fluctuations, while the latter suggests that plants tend to resorb the more limiting nutrient preferentially [23,29,30,31]. Our results align with observations of *P. sylvestris* in European and North American temperate forests, where foliar N and P concentrations are typically maintained within specific physiological thresholds to support metabolic functions [32]. Recent investigations into European Scots pine populations (Poland and the Mediterranean) have highlighted that under the dual pressures of climate warming and soil nutrient imbalances, trees increasingly prioritize internal nutrient cycling to sustain metabolic activity [33,34].

In the nutrient–limited environment of the Otindag Sandy Land, *P. sylvestris* prioritizes nutrient stability in its apical needles to ensure continuous growth. This aligns with the principle of ecological stoichiometric homeostasis, which dictates that active growth centers must maintain an optimal elemental composition to sustain metabolism under stress [23]. In contrast, older basal needles exhibit greater stoichiometric plasticity and function as transient nutrient reservoirs. Utilizing these older organs is a recognized adaptive strategy, allowing plants to actively up-regulate nutrient resorption to mitigate resource limitation and enhance drought resistance [35,36]. In the observed chlorotic trees, the intensified reallocation of N and P successfully safeguards the apical needles. However, this massive nutrient export directly triggers a stoichiometric collapse in the basal needles. This active physiological regulation exemplifies a targeted survival strategy. Under severe stress, plants deliberately sacrifice subordinate organs to protect vital developing tissues and recycle essential nutrients [37]. Consequently, basal needle chlorosis is not merely a passive mass transfer. Instead, it represents a regulated functional shift, transforming these needles from stable carbon–assimilating organs into terminal nutrient-supplying pools within the tree’s overall adaptive strategy.

In addition to N and P, deficiencies of other key elements such as magnesium (Mg) and iron (Fe) can also lead to needle chlorosis. Research on *Pinus taeda* L. in Brazil showed that Mg concentrations in chlorotic needles were well below critical thresholds—only one-third of the required level [38]. As the central atom of the chlorophyll molecule, Mg deficiency severely reduces chlorophyll content, allowing yellow and orange carotenoid pigments to become visible, thereby resulting in chlorosis. Unlike the overall needle chlorosis in this study, Mg deficiency typically manifests as interveinal chlorosis, where veins remain green while interveinal tissues turn yellow [39,40]. In contrast, iron is not a structural component of chlorophyll but is essential for its synthesis [41]. Thus, Fe deficiency also leads to chlorosis due to impaired chlorophyll production. However, Fe has low mobility within plants and cannot be effectively translocated from older leaves to actively growing apical meristems and young leaves [42]. Although Mg and Fe concentrations were not directly measured in the current study, based on the distinct visual symptom patterns documented in previous literature, deficiencies of these elements are less likely to be the primary drivers of needle chlorosis in the *P. sylvestris* plantations of the Otindag sandy land. Instead, the coordinated reallocation and balance of N and P appear to play a more critical role in the observed chlorosis pattern.

### 4.2. Decoupling Between Branch Basal Needle Chlorosis and Soil Nutrient Availability

Classical understanding of tree decline, including needle chlorosis, has largely attributed the phenomenon to soil nutrient deficiency. This paradigm has been further reinforced by recent large–scale assessments spanning diverse ecosystems. Work by Prietzel et al. [34] in Southern Germany forests demonstrated that long term variations in soil N and P availability remain the primary determinants of foliar chemistry in *P. sylvestris*. Together with findings from North American boreal ecosystems, where nutrient uptake and foliar concentrations remain strictly coupled with soil fertility, these serve as critical indicators of forest resilience to environmental shifts [43]. Collectively, insufficient availability of nutrients in the soil directly limits root uptake, leading to reduced concentrations of key elements such as N and P in the needles [44]. This deficiency subsequently impairs chlorophyll biosynthesis, disrupts key metabolic functions, and ultimately leads to visible chlorosis—a view substantiated by extensive evidence from studies across varied forest ecosystems [45,46,47]. This pattern also holds true in sandy land ecosystems, where nutrient limitations are often pronounced [5,48,49]. It is important to clarify that a lower SDI does not imply a small overall effect of the soil, as the chronically nutrient—poor soil is the fundamental driver of the observed stress. Rather, a lower SDI indicates a strong physiological decoupling between needle and soil stoichiometric ratios. This highlights the tree’s capacity for active internal homeostatic regulation—specifically the prioritized reallocation of nutrients—to maintain functional stability despite the extreme imbalances in the external soil environment.

However, the analysis of deviations in C:N and C:P in this study provides complementary and inconsistent evidence. Both indicators reveal a severe mismatch between the nutrient status of chlorotic needles and soil nutrient availability, with this decoupling phenomenon persisting across all soil layers. Similar patterns have been observed in *P. sylvestris* and *P*. *alba* ecosystems in the Horqin Sandy Land [13,14], as well as in shrublands of the Hengduan Mountains [15]. From the perspective of two key limiting elements, N and P, these results are inconsistent with soil nutrient deficiency as the primary cause and instead point to internal nutrient reallocation within the tree as the driver of needle chlorosis. In addition to C:N and C:P deviations, analysis of the N:P deviation also indicates a degree of disconnection between senescing basal needles and soil nutrient balance, as reflected in the soil deviation index (*SDI* = 0.73 in the 0–20 cm layer). However, compared to the extreme deviations observed for C:N (11.6) and C:P (16.1), the N:P deviation is relatively moderate. This may be explained by the fact that the N:P ratio is concurrently influenced by both N and P concentrations; in chlorotic needles, both elements are substantially reallocated, resulting in a less pronounced shift in their ratio relative to carbon based indices. Nevertheless, all three deviation metrics consistently support the same conclusion: the nutrient status of chlorotic needles is governed principally by internal reallocation processes rather than by soil nutrient supply.

### 4.3. Limitations and Perspectives

While this study provides novel stoichiometric insights into plantation decline, several inherent limitations should be noted. First, although an *n* = 3 is acceptable for capturing strong biological signals in exploratory ecophysiological studies, it limits the statistical power of multivariate analyses (e.g., PCA) and the reliability of Pearson correlations. Consequently, variance–based stoichiometric indices (such as CV, HI, and SDI) and the identified trends should be interpreted cautiously as preliminary physiological indicators and potential associations. Furthermore, our physiological inferences rely primarily on stoichiometric proxies. The absence of direct physiological measurements, such as chlorophyll content, gas exchange rates, and micro nutrient concentrations (e.g., Mg and Fe), represents a limitation for a comprehensive physiological validation. The NRE calculated in this study must be interpreted within a specific physiological context. Because the basal chlorotic needles had not reached the absolute terminal senescence stage, the NRE here functions more accurately as a quantitative index of premature, stress induced nutrient mobilization. Nevertheless, this distinction perfectly aligns with our hypothesis of an active, stress driven optimal partitioning strategy within the tree. Given these constraints, the current study is fundamentally preliminary and exploratory. Future research should expand the sample size across broader spatial scales and incorporate controlled experiments with direct physiological and micro–nutrient monitoring. Such comprehensive approaches are required to fully elucidate the underlying physiological drivers of needle chlorosis. Furthermore, they are essential to statistically validate the internal nutrient reallocation patterns observed in this study.

## 5. Conclusions

This study reveals that the chlorosis observed in branch basal needles of *P. sylvestris* in the Otindag Sandy Land is closely associated with the internal reallocation of nitrogen (N) and phosphorus (P) to support apical growth, rather than being directly coupled with immediate soil nutrient deficiency. The basal needles of chlorotic trees show significant co–limitation by N and P, with elevated C:N and C:P ratios, indicating nutrient imbalance in these organs. Moreover, the decoupling between needle chlorosis and soil nutrient availability provides a new stoichiometric perspective on the physiological underpinnings behind plantation degradation in sandy ecosystems. These findings highlight the importance of internal nutrient cycling in tree adaptation to sandy ecosystems and offer practical insights for the management and restoration of degrading plantations.

## Data Availability

Zhang. (2026). raw data [Data set]. Zenodo. https://doi.org/10.5281/zenodo.18136353.

## References

[1] Kono A., Kimura K., Yamada S., Koyanagi T.F., Yamanaka N., Yoshikawa K., Tsuchiya K., Okuro T. (2025). Effectiveness of sand-fixing measures for restoration of vegetation and mitigation of wind erosion and deposition in a degraded sandy rangeland, northern China. Ecol. Eng..

[2] Kurmangozhinov A., Xue W., Li X., Zeng F., Sabit R., Tusun T. (2020). High biomass production with abundant leaf litterfall is critical to ameliorating soil quality and productivity in reclaimed sandy desertification land. J. Environ. Manag..

[3] Allen C.D., Macalady A.K., Chenchouni H., Bachelet D., McDowell N., Vennetier M., Kitzberger T., Rigling A., Breshears D.D., Hogg E.H. (2010). A global overview of drought and heat-induced tree mortality reveals emerging climate change risks for forests. For. Ecol. Manag..

[4] Hevia A., Sánchez-Salguero R., Camarero J.J., Querejeta J.I., Sangüesa-Barreda J., Gazol A. (2019). Long-term nutrient imbalances linked to drought-triggered forest dieback. Sci. Total Environ..

[5] Bi B., Wang Y., Wang K., Zhang H., Fei H., Pan R., Han F. (2022). Changes in microbial metabolic C- and N- limitations in the rhizosphere and bulk soils along afforestation chronosequence in desertified ecosystems. J. Environ. Manag..

[6] Schulze E.D. (1989). Air pollution and forest decline in a spruce (*Picea abies*) forest. Science.

[7] Wang P., Zhu L., Wang X., Zhai Y., Zhong J., Zhang H., Shao J., Liu X., Yu F., Qi Y. (2025). Balance of chlorophyll synthesis and thylakoid protein quality control influences chloroplast development in var2 mutants. J. Exp. Bot..

[8] Li X., Zhang W., Niu D., Liu X. (2024). Effects of abiotic stress on chlorophyll metabolism. Plant Sci..

[9] Sakuraba Y. (2022). Molecular basis of nitrogen starvation-induced leaf senescence. Front. Plant Sci..

[10] Liu W., Sun Q., Wang K., Du Q., Li W. (2017). Nitrogen Limitation Adaptation (NLA) is involved in source-tosink remobilization of nitrate by mediating the degradation of NRT1.7 in Arabidopsis. New Phytol..

[11] Khan F., Siddique A.B., Shabala S., Zhou M., Zhao C. (2023). Phosphorus plays key roles in regulating plants’ physiological responses to abiotic stresses. Plants.

[12] Poirier Y., Jaskolowski A., Clúa J. (2022). Phosphate acquisition and metabolism in plants. Curr. Biol..

[13] Wang K., Wang G.G., Song L., Zhang R., Yan T., Li Y. (2021). Linkages Between Nutrient Resorption and Ecological Stoichiometry and Homeostasis Along a Chronosequence of Mongolian Pine Plantations. Front. Plant Sci..

[14] Wang K., Zhang R., Song L., Yan T., Na E. (2021). Comparison of C:N:P Stoichiometry in the Plant–Litter–Soil System Between Poplar and Elm Plantations in the Horqin Sandy Land, China. Front. Plant Sci..

[15] Li L., Chen D., Huang X., Liu Q., Liang J., Hu J., Liu Q. (2024). Variations of nitrogen and phosphorus between leaf, stem and root in shrubland biomes and responses to climate and soil factors across the Hengduan Mountains, China. Catena.

[16] Wang L., Ju C., Han C., Yu Z., Bai M., Wang C. (2025). The interaction of nutrient uptake with biotic and abiotic stresses in plants. J. Integr. Plant Biol..

[17] Mishra S., Levengood H., Fan J., Zhang C. (2024). Plants Under Stress: Exploring Physiological and Molecular Responses to Nitrogen and Phosphorus Deficiency. Plants.

[18] Estiarte M., Campioli M., Mayol M., Penuelas J. (2023). Variability and limits of nitrogen and phosphorus resorption during foliar senescence. Plant Comm..

[19] Kobe R.K., Iyer M., Walters M.B. (2010). Optimal partitioning theory revisited: Nonstructural carbohydrates dominate root mass responses to nitrogen. Ecology.

[20] Ma Y., Huang N., Ma C. (2023). Heterogeneity, marginality, stagementation and driving forces in the Otindag Sandy Land and its ecotones based on GIMMS NDVI3g v1.0. Ecol. Inform..

[21] Gan H., Yang H., Chu J., Li Y., Fang K., Zhang Q., Sun J. (2026). Stoichiometry and drivers of soil C, N, P and K in the Otindag Sandy Land, northern China. Plant Soil.

[22] Bao S. (2000). Soil and Agricultural Chemistry Analysis.

[23] Su B., Shangguan Z. (2022). Stoichiometric homeostasis in response to variable water and nutrient supply in a *Robinia pseudoacacia* plant–soil system. J. Plant Ecol..

[24] Reich P.B., Oleksyn J. (2004). Global patterns of plant leaf N and P in relation to temperature and latitude. Proc. Natl. Acad. Sci. USA.

[25] Chen X., Chen H.Y.H. (2021). Plant mixture balances terrestrial ecosystem C:N:P stoichiometry. Nat. Commun..

[26] Tian H., Chen G., Zhang C., Melillo J.M., Hall C.A.S. (2010). Pattern and variation of C:N:P ratios in China’s soils: A synthesis of observational data. Biogeochemistry.

[27] Yu M., Zhaxi L., Deqing Z., Wei X., Tang Y. (2025). Advances in plant response to low-temperature stress. Plant Growth Regul..

[28] Zia R., Nawaz M.S., Siddique M.J., Hakim S., Imran A. (2021). Plant survival under drought stress: Implications, adaptive responses, and integrated rhizosphere management strategy for stress mitigation. Microbiol. Res..

[29] López-Sepulcre A., Amaral J.R., Gautam N., Mohamed A., Naik S. (2024). The eco-evolutionary dynamics of stoichiometric homeostasis. Trends Ecol. Evol..

[30] Freschet G.T., Cornelissen J.H.C., van Logtestijn R.S.P., Aerts R. (2010). Substantial nutrient resorption from leaves, stems and roots in a subarctic flora: What is the link with other resource economics traits?. New Phytol..

[31] Pornon A., Lamaze T. (2007). Nitrogen resorption and photosynthetic activity over leaf life span in an evergreen shrub, *Rhododendron ferrugineum*, in a subalpine environment. New Phytol..

[32] Oleksyn J., Reich P.B., Zytkowiak R., Karolewski P., Tjoelker M.G. (2003). Nutrient conservation increases with latitude of origin in European *Pinus sylvestris* populations. Funct. Ecol..

[33] Sardans J., Peñuelas J. (2021). Potassium control of plant functions: Ecological and evolutionary implications. Plants.

[34] Prietzel J., Stetter U. (2010). Long-term trends of phosphorus nutrition and topsoil phosphorus stocks in unfertilized and fertilized Scots pine (*Pinus sylvestris*) stands at two sites in Southern Germany. Forest Ecol. Manag..

[35] Zhang S., Song Y., Wen H., Chen Y. (2024). Leaf nitrogen and phosphorus resorption efficiencies are related to drought resistance across woody species in a Chinese savanna. Tree Physiol..

[36] Wang P., Fu C., Wang L., Yan T. (2022). Delayed autumnal leaf senescence following nutrient fertilization results in altered nitrogen resorption. Tree Physiol..

[37] Sasi J.M., Gupta S., Singh A., Kujur A., Agarwal M., Katiyar-Agarwal S. (2022). Know when and how to die: Gaining insights into the molecular regulation of leaf senescence. Physiol. Mol. Biol. Plants.

[38] Motta A.C.V., Maeda S., Rodrigues V.D.S., Ercole T.M., Prior S.A., Brumat A.E.L., Moura A.P.C., Barbosa J.Z., Gomes J.B.V. (2024). Is magnesium deficiency the major cause of needle chlorosis of *Pinus taeda* in Brazil?. J. For. Res..

[39] Guo W., Nazim H., Liang Z., Yang D. (2016). Magnesium deficiency in plants: An urgent problem. Crop J..

[40] Gransee A., Führs H. (2013). Magnesium mobility in soils as a challenge for soil and plant analysis, magnesium fertilization and root uptake under adverse growth conditions. Plant Soil.

[41] Zheng S. (2010). Iron homeostasis and iron acquisition in plants: Maintenance, functions and consequences. Ann. Bot..

[42] Rai S., Singh P.K., Mankotia S., Swain J., Satbhai S.B. (2021). Iron homeostasis in plants and its crosstalk with copper, zinc, and manganese. Plant Stress.

[43] Yuan Z.Y., Chen H.Y.H., Reich P.B. (2011). Global-scale latitudinal patterns of plant fine-root nitrogen and phosphorus. Nat. Commun..

[44] Craig M.E., Walker A.P., Iversen C.M., Knox R.G., Yaffar D., York L.M. (2025). Tree root nutrient uptake kinetics vary with nutrient availability, environmental conditions, and root traits: A global analysis. New Phytol..

[45] Yang Z., Mao Z., Ji W., Gazol A., Liu S., Wang C., Ye J., Lin F., Wang X., Hao Z. (2025). Nitrogen addition accelerates aboveground biomass sequestration in old-growth forests by stimulating ectomycorrhizal tree growth. J. Environ. Manag..

[46] Tang F., Zhou Y., Deng P., Feng J., Mao Y., Wang Y., Cao Q., Han Z., Meng L., Bai Y. (2024). Soil phosphorus compared to nitrogen limitation increases the uncertainty of subsoil organic carbon sequestration in *Pinus massoniana* mixed forests. J. Environ. Manag..

[47] Siah K.G., Perakis S.S., Pett-Ridge J.C., van der Heijden G. (2023). Nitrogen-bedrock interactions regulate multi-element nutrient limitation and sustainability in forests. Biogeochemistry.

[48] Cheng L., Zhan J., Ning Z., Li Y. (2025). Influence of nitrogen inputs on biomass allocation strategies of dominant plant species in sandy ecosystems. J. Arid Land.

[49] Drenovsky R.E., Richards J.H. (2005). Nitrogen addition increases fecundity in the desert shrub *Sarcobatus vermiculatus*. Oecologia.

